# Antioxidant Response in Human X-Linked Adrenoleukodystrophy Fibroblasts

**DOI:** 10.3390/antiox11112125

**Published:** 2022-10-28

**Authors:** Sara Petrillo, Jessica D’Amico, Francesco Nicita, Caterina Torda, Gessica Vasco, Enrico S. Bertini, Marco Cappa, Fiorella Piemonte

**Affiliations:** 1 Unit of Muscular and Neurodegenerative Diseases, Bambino Gesù Children’s Hospital, IRCCS, 00146 Rome, Italy; 2 Movement Analysis and Robotics Laboratory (MARLab), Department of Neurorehabilitation and Robotics, Bambino Gesù Children’s Hospital, IRCCS, 00050 Rome, Italy; 3 Unit of Endocrinology, Bambino Gesù Children’s Hospital, IRCCS, 00146 Rome, Italy

**Keywords:** X-ALD, AMN, cALD, lipid peroxidation, glutathione peroxidase 4, NAD(P)H coenzyme Q oxidoreductase 1, glutathione, ferroptosis, oxidative stress

## Abstract

Redox imbalance, mitochondrial dysfunction, and inflammation play a major role in the pathophysiology of X-linked adrenoleukodystrophy (X-ALD), an inherited neurodegenerative disease caused by mutations in the ABCD1 gene, encoding the protein responsible for peroxisomal import and degradation of very long chain fatty acids (VLCFAs). Therefore, VLCFAs accumulate in tissues and plasma, constituting a pathognomonic biomarker for diagnosis. However, the precise role of VLCFA accumulation on the diverse clinical phenotypes of X-ALD and the pathogenic link between VLCFAs and oxidative stress remain currently unclear. This study proposes ferroptosis as a crucial contributor to the disease development and progression. The expression profiles of “GPX4-glutathione” and “NQO1-CoQ_10_” ferroptosis pathways have been analyzed in fibroblasts of one patient with AMN, the late onset and slowly progressive form of X-ALD, and in two patients with cALD, the cerebral inflammatory demyelinating form of early childhood. Furthermore, as no effective treatments are currently available, especially for the rapidly progressing form of X-ALD (cALD), the efficacy of NAC treatment has also been evaluated to open the way toward novel combined therapies. Our findings demonstrate that lipid peroxides accumulate in X-ALD fibroblasts and ferroptosis-counteracting enzymes are dysregulated, highlighting a different antioxidant response in patients with AMN and cALD.

## 1. Introduction

Oxidative stress is the major contributor to the pathogenesis of X-linked adrenoleukodystrophy (X-ALD) [1,2,3,4,5,6], an inherited neurodegenerative disease caused by mutations of the ABCD1 gene, encoding a peroxisomal transmembrane protein responsible for peroxisomal import and degradation of very-long-chain fatty acids (VLCFAs) [7,8]. As a consequence, VLCFAs accumulate in tissues and plasma, thus constituting a pathognomonic biomarker for diagnosis [9]. However, although the increase of VLCFA in plasma and tissues of patients represents the diagnostic marker of X-ALD, VLCFAs overload does not necessarily reflect the disease severity, and the relationship between VLCFAs accumulation and the disease development/progression is still unknown.

Under condition of oxidative stress, an abnormal lipid metabolism may cause lipid peroxidation and triggers ferroptosis, a new mechanism of oxidative-mediated cell death leading to membrane dysfunction and, ultimately, neuro-inflammation and neuodegeneration [10,11].

Previous studies reported redox imbalance in plasma, red blood cells, skin fibroblasts, and nervous tissues of patients with X-ALD, highlighting oxidative stress as the early pathogenic factor of the disease [1,2,3,4,12,13,14,15]. In this study, we used X-ALD primary fibroblasts obtained from one patient with AMN, the late onset and slowly progressive form of X-ALD, and two patients with cALD, the cerebral inflammatory demyelinating form of early childhood, to investigate the expression of the main biochemical hallmarks of antioxidant response, particularly focusing on the ferroptosis-related biomarkers. 

Fibroblasts are a good cell model, because they recapitulate the main disease hallmarks, such as VLCFA accumulation [16], free radicals overload [3], energetic impairment [17], defective proteostasis [18], and endoplasmic reticulum stress [19]. We analyzed proteins mainly involved in the antioxidant protection against membrane lipid peroxidation. Specifically, we focused on the glutathione peroxidase 4 (GPX4), which converts lipid peroxides to their corresponding non-toxic alcohols; the glutathione reductase (GR), regenerating the active form of glutathione (GSH) for a steady cell supply; the NAD(P)H:quinone oxidoreductase-1 (NQO1) that detoxifies quinones to hydroquinones, thus insuring a correct membranes redox balance [20]. These proteins work in concert for the protection against lipotoxicity, thus understanding their modulation in different X-ALD clinical phenotypes can provide novel insights into the pathogenic mechanism linking VLFCAs accumulation and oxidative damage. Furthermore, as no treatment is currently available for the AMN phenotype, although a phase II pilot trial using a combination of high-dose antioxidants seems promising [21], here we used X-ALD patient’s fibroblasts to investigate the preclinical efficacy of N-acetyl cysteine (NAC) on antioxidant response and lipid peroxidation, in order to identify new therapeutic options.

## 2. Materials and Methods

### 2.1. Fibroblasts Cultures

Skin biopsies were taken from n. 3 clinically affected (and genetically proven) X-ALD patients (two cALD and one AMN) and n. 5 age-matched controls (Ctrls). Fibroblasts were grown in Dulbecco’s modified Eagle’s medium supplemented with 10% fetal bovine serum, 50 units/mL penicillin, 50 µg/mL streptomycin, 0.4% (*v*/*v*) amphotericin B (250 μg/mL), at 37 °C in 5% CO_2_.

C22:0 (docosanoic acid), C24:0 (tetracosanoic acid), and C26:0 (hexacosanoic acid) content were determined in fibroblasts by a high performance liquid chromatography-tandem mass spectrometry (HPLC-MS/MS) method (as reported in Semeraro et al., 2016) [22], using an API3200 triple quadrupole mass spectrometer (Applied Biosystems-MDS Sciex, Toronto, Canada), equipped with an APCI source operating in negative ion mode with a nebulizer current of −3 and a source temperature of 500 °C (Table 1).

For the treatment, cells were cultured to 70% confluence and incubated 24 h with 100 µM N-acetyl cysteine (NAC) (drug dosing has been chosen following Petrillo et al., 2019, [23]). After washing, cells were lysed in Total RNA Purification Plus Kit (Norgen Biotek Corp., Torold, ON, Canada), according to the manufacturer’s protocol for RNA extraction and subjected to quantitative real-time PCR, or lysed with RIPA buffer including DTT and protease inhibitors for Western blotting analyses. Cells were used at similar 9–11 passage numbers and tested to exclude mycoplasma contamination. The assays were performed in triplicates. All participants signed an informed consent and the study was approved by the Ethics Committee of “Bambino Gesù” Children’s Hospital.

### 2.2. Assessment of Lipid Peroxidation by C11-Bodipy (581/591) Fluorescent Staining

Fibroblasts were incubated with 5 µM C11-BODIPY581/591 (D3861, Thermo Scientific, Waltham, MA, USA) for 30 min at 37 °C and images were acquired with a Leica DMi8 fluorescence microscope (Leica Microsystems, Wetzlar, Germany). Stock solutions were made by dissolving 1 mg C11-BODIPY581/591 in 50 μL dimethyl sulfoxide (DMSO).

### 2.3. Quantitative Real Time PCR (qRT-PCR)

About 1 µg RNA samples was reverse transcribed and expression levels of GPX4, GCL, GR, and NQO1 were measured as previously reported [23]. Data were analyzed by the 2-DDCt method using TBP (TATA box binding protein) as housekeeping gene.

### 2.4. Western Blot Analysis

Fibroblasts (1 × 10^6^) lysate containing 40 µg proteins was subjected to SDS PAGE on 4–12% denaturing gel as described in Petrillo et al. [23]. The following antibodies have been used: Gpx4 (1:500, RD Systems, Biotechne Brand, MN, USA), Gr (1:1000, Novus Biological, CO, USA), Nqo1 (1:5000. Novus Biological, CO, USA), and GAPDH (1:10,000, Sigma Aldrich, St. Louis, MO, USA) as loading control. Proteins were quantified by Pierce™ BCA Protein Assay Kit (Thermo Scientific, Waltham, MA, USA).

### 2.5. NQO1 Enzyme Activity Assay

Dicoumarol-sensitive NQO1 activity was measured on fibroblasts by following the absorbance decrease at 600 nm due to the reduction of the substrate 2,6-dichloro-indophenol (DCIPIP). About 10 µg proteins were added to the reaction mix consisting of 25 mM Tris–HCl, pH 7.4, 0.18 mg/mL BSA, 5 mM FAD, 0.01% Tween 20, 200 mM NADH, and 50 mM DCPIP. The reaction was monitored for 1 min at 25 °C on a double beam spectrophotometer Lambda 365 (Perkin Elmer). To determine the specific activity of NQO1, an extinction coefficient of 21 mM/cm was used and protein content was quantified by the BCA method (ThermoFisher, USA).

### 2.6. GPX4 Enzyme Activity Assay

GPX4 assay was performed on fibroblasts using cumene hydroperoxide as GPX4-specific substrate and glutathione reductase for GSSG reduction. About 50 µg proteins were added to the reaction mix containing 100 mM Tris, pH 7.8, 5 mM EDTA, 0.1% Triton X-100, 3 mM GSH, 0.2 mM NADPH, 0.6 U/mL glutathione reductase, and 20 µM cumene hydroperoxide. The GPX4 activity was measured following the decrease of NADPH absorbance at 340 nm by an EnSpire Multimode Plate Reader (Perkin Elmer, Waltham, MA, USA).

### 2.7. GSH Assay

Glutathione levels have been detected in fibroblasts by an enzymatic re-cycling assay [23]. Samples de-proteinization was carried out using 5% (*w*/*v*) sulphosalycilic acid (SSA, Sigma-Aldrich, St. Louis, MO, USA). The acid soluble fraction was diluted in Na-phosphate buffer containing EDTA (pH 7.5) to determine the glutathione content. Thiols (SH) and GSH levels were assessed by the ThioStar^®^ glutathione detection reagent (Arbor Assays, Michigan, MI, USA) and the fluorescence was measured by an EnSpire^®^ Multimode Plate Reader (Perkin Elmer, Waltham, MA, USA). 

### 2.8. Statistical Analysis

Statistical analysis was performed using GRAPHPAD/Prism 5.0 Software (GraphPad Company, San Diego, CA, USA). Statistically significant differences between groups were analyzed using Student’s t-test for normally distributed variables. All data are presented as mean ± standard error. Statistical significance was defined as * *p* < 0.05, ** *p* < 0.01, *** *p* < 0.001 compared to healthy controls, and # *p* < 0.05, ## *p* < 0.01, ### *p* < 0.001 compared to untreated cells.

## 3. Results

### 3.1. The Antioxidant Response Is Differently Modulated in Fibroblasts of Patients with AMN and cALD

**Figure 1:**
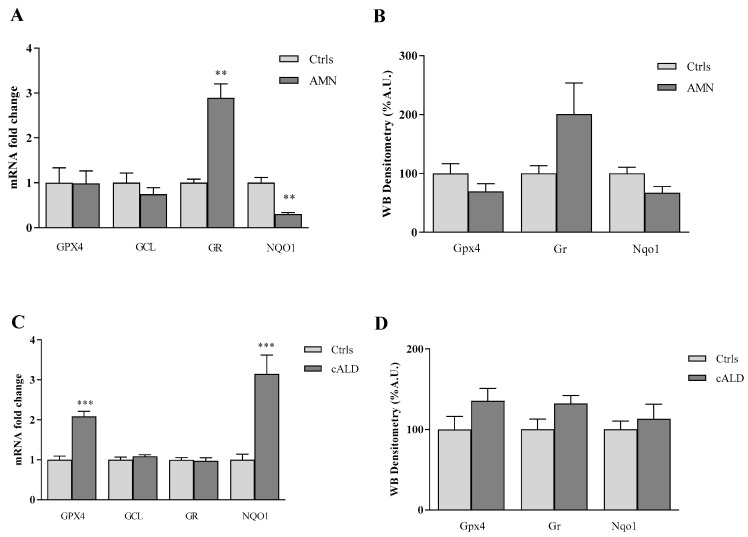
Antioxidant enzymes expression in fibroblasts of patients with AMN (**A**,**B**) and cALD (**C**,**D**). (**A**,**C**) qRT-PCR analyses of GPX4, GCL, GR, NQO1 mRNA transcripts. (**B**,**D**) Densitometric analysis of Gpx4, Gr and Nqo1 protein levels. Experiments were conducted in triplicates and values expressed as mean ± SEM. ** *p* < 0.01, *** *p* < 0.001, compared to control’s group.

Under physiological conditions, the cellular redox and lipid homeostasis is kept in balance by a complex defense system, including glutathione (GSH), glutathione peroxidase 4 (GPX4), NADPH:quinone oxidoreductase (NQO1), and glutathione reductase (GR). To understand if a different modulation of these antioxidant proteins occurs in patients with several forms of X-ALD, we analyzed their expression in fibroblasts of patients with AMN, the late onset and slowly progressive form of X-ALD, and cALD, the severe cerebral form of early childhood. As reported in Figure 1, the analysis of gene expression shows a significant decrease (70%) of NQO1 in the patient with AMN, while consistent increases of GR (2.9-fold) transcripts have been found by qRT-PCR (Figure 1A, Table 2). Western blot analysis confirmed the increase (2-fold) of GR and the decrease (33%) of NQO1 protein amounts (Figure 1B, Table 2), thus indicating an activation of the GSH-recycling enzyme (GR) in AMN cells, whereas the enzyme responsible for the CoQ_10_ antioxidant response (NQO1) and lipid protection was reduced as well.

Unlike AMN, fibroblasts derived from patients with cALD (Figure 1C, Table 2) show a consistent activation of GPX4 and NQO1 antioxidant genes, either as transcripts (2-fold GPX4, 3-fold NQO1, Figure 1C), and as protein levels (36% GPX4, 13% NQO1, 32% GR increases, Figure 1D, Table 2), although not reaching, the latter, the statistical significance. These data highlight a consistent up-regulation of the antioxidant defense provided by GPX4 and NQO1 in cALD, indicating the need in these cells to promptly respond to lipid oxidative damage. 

### 3.2. GPX4, NQO1 and GSH: A Pathological Triad in X-ALD?

**Figure 2:**
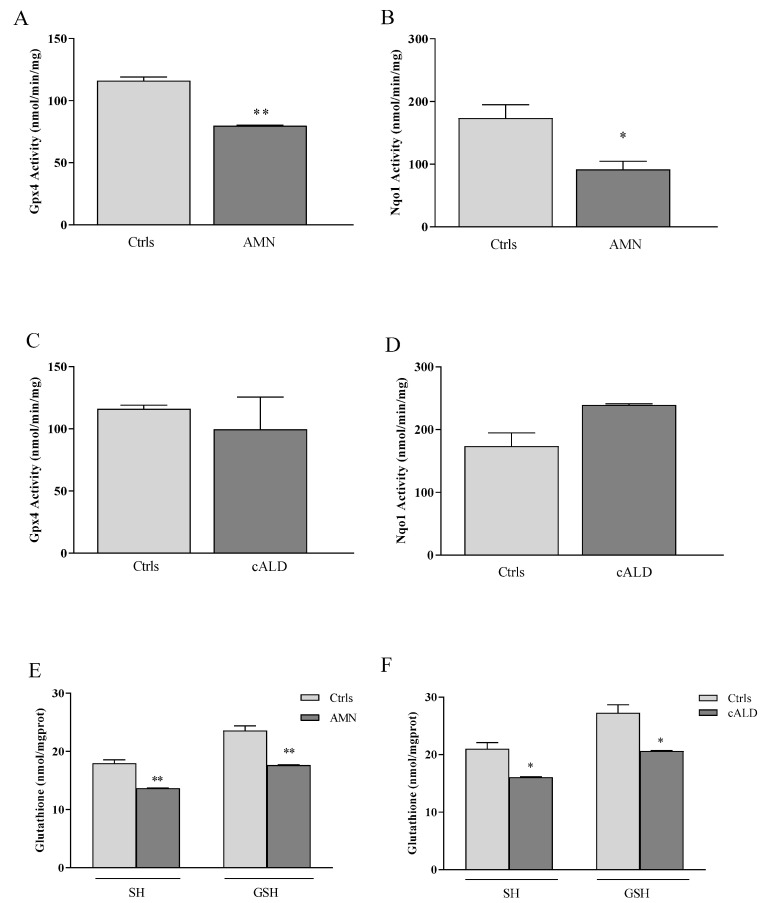
(**A**,**C**) Gpx4 and (**B**,**D**) Nqo1 enzyme activities. (**E**,**F**) Thiols and glutathione (GSH) content. Experiments were performed in triplicates and values expressed as mean ± SD. * *p* < 0.05, ** *p* < 0.01, compared to controls (by Student’s two-tailed *t*-test).

GPX4 and NQO1 are essential to maintaining lipid homeostasis in cells. GPX4 catalyzes the detoxification of lipid peroxides into nontoxic lipid alcohols, and NQO1 maintains the membrane redox balance by converting the oxidized form of CoQ_10_ to the reduced/active one (CoQH_2_). Thus, to go deeper into the protein function, we measured the enzyme activities of GPX4 and NQO1 in AMN and cALD fibroblasts (Figure 2). We found a significant decrease in both GPX4 (32%) and NQO1 (47%) enzyme activities in AMN (Figure 2A,B, Table 3), thus confirming the low protein contents observed by Western blotting (Figure 1B). Conversely, and in line with the up-regulation of its transcript (Figure 1C), NQO1 was significantly activated (38%, D) in cALD, while the GPX4 activity, despite high mRNA levels (Figure 1C), was not increased (Figure 2C,D, Table 3).

It is important to underline that GPX4 needs GSH as enzyme cofactor, to best perform its activity. Therefore, we measured GSH concentrations in fibroblasts of patients and evidenced 25% and 24% GSH decreases in fibroblasts of patients with AMN (Figure 2E, Table 3)) and cALD (Figure 2F, Table 3)), respectively. Nevertheless, the enzyme γ-glutamylcysteine ligase (GCL), responsible for the de novo synthesis of GSH, was not induced either in AMN or in cALD cells (Figure 1), indicating failure in rebalancing the GSH pool. To note, besides its essential role in GPX4 activity, GSH is the primary ROS scavenger in cells, thus its dysregulation can further compromise the antioxidant response in this disease. 

### 3.3. Lipid Peroxidation Increases in Patients with X-ALD

**Figure 3:**
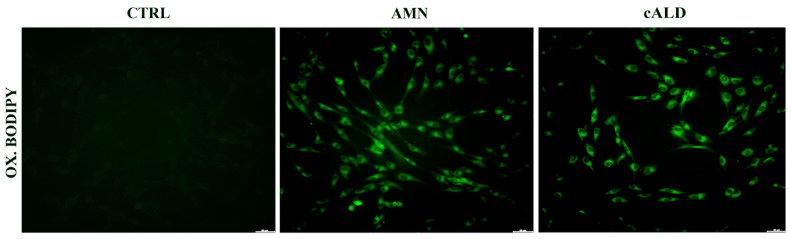
Representative images of lipid peroxides staining (green), detected by the fluorescent lipophilic Bodipy-C11 sensor, of X-ALD patients’ fibroblasts (scale bar = 100 μm).

GPX4, NQO1, and GSH are essential to counteract the formation of lipid peroxides in the biological membranes, thus we analyzed lipid peroxidation in X-ALD fibroblasts by following the increase of C-11 Bodipy (581/591) green fluorescence in cultured cells [24]. As reported in Figure 3, lipid peroxides consistently accumulate in both AMN and cALD fibroblasts, probably as a consequence of a compromised antioxidant response associated to lipid dysregulation. 

### 3.4. The Effect of NAC on the Antioxidant Response in Fibroblasts of Patients with X-ALD

**Figure 4:**
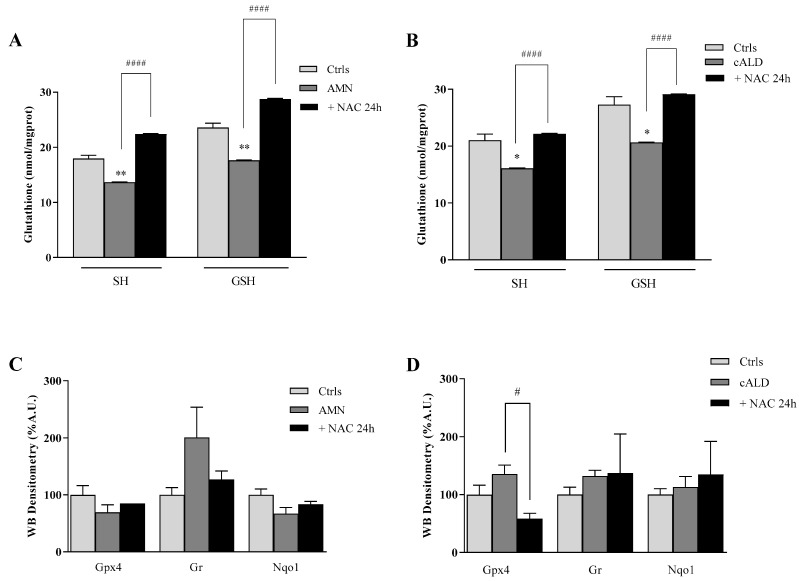
Antioxidant enzymes expression in fibroblasts of patient with X-ALD after 24 h treatment with NAC (100 µM). (**A**,**B**) Thiols and Glutathione (GSH) content. (**C**,**D**) Densitometric analysis of Gpx4, Gr, and Nqo1 protein levels. Experiments were performed in triplicates and values expressed as mean ± SD. * *p* < 0.05, ** *p* < 0.01, compared to controls (by Student’s two-tailed *t*-test). # *p* < 0.05, #### *p* < 0.001, respect to untreated patient’s fibroblasts.

**Figure 5:**
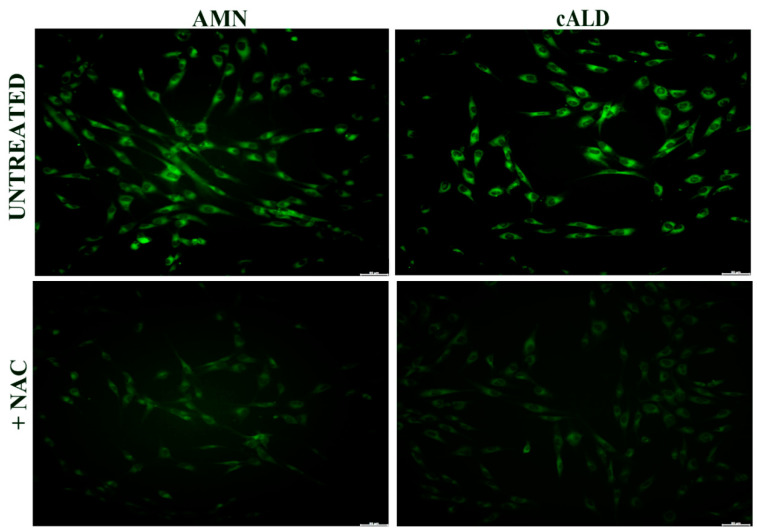
Representative images of Lipid peroxides staining (green), detected by using the fluorescent lipophilic Bodipy-C11 sensor, of X-ALD fibroblasts after 24 h treatments with NAC (100 µM, scale bar = 100 μm).

Currently, no treatments are available for the AMN phenotype, but a phase II pilot trial provided promising results using high doses of antioxidants [21]. Moving from this evidence, we incubated AMN and cALD fibroblasts with NAC, to evaluate its effect on the antioxidant response and on lipid peroxides accumulation. NAC is a powerful reducing agent with a crucial role as co-substrate in the GSH synthesis. As expected, the amount of GSH significantly increased after 24 h NAC treatment, either in AMN (61%) or in cALD (41%) (Figure 4A,B, Table 4). Consequently, the enzyme GR, which is responsible for the regeneration of GSH from GSSG and was up-regulated in AMN (Figure 1A), decreased to levels comparable to controls after treatment with NAC (Figure 4C, Table 4), except in patient 2 where protein levels were high (Figure 4D, Table 4). Conversely, GPX4 and NQO1 appeared only slightly increased in AMN (Figure 4C, Table 4) while, in cALD, the GPX4 expression was significantly decreased after 24 h NAC treatment with respect to untreated cells (Figure 4D, Table 4) and NQO1 showed an individual response: not affected in cALD 1 and up-modulated in cALD 2 (Figure 4D, Table 4). Interestingly, lipid peroxidation significantly decreased after NAC incubation, as detected by the quenching of C-11 Bodipy (581/591) green fluorescence (Figure 5). These findings support a direct effect of NAC on the GSH homeostasis and highlight an indirect efficacy on lipid peroxidation, paving the way to combined-synergistic therapeutic approaches.

## 4. Discussion

Under conditions of oxidative stress, an abnormal lipid metabolism may cause lipid peroxidation, membrane dysfunction and ferroptosis, ultimately leading to neuro-inflammation and neurodegeneration [10,11]. Oxidative stress is considered the early culprit in X-ALD [1,25,26], a clinically heterogeneous disease characterized by progressive central nervous system (CNS) demyelination, spinal cord axonopathy, and adrenal insufficiency [27,28]. Three phenotypes may be observed in patients: an isolated adrenal insufficiency (i.e., Addisons’s disease), a late onset slowly progressive adrenomyeloneuropathy (AMN), and a cerebral inflammatory demyelinating form (cALD), which can be fatal in early childhood [27,29].

The same ABCD1 mutation may lead to cALD, AMN, or Addison’s disease, either in different individuals [30] and also in genetically identical twins [31,32], thus proving that genetic background is not the only determinant of the phenotypic heterogeneity in this disease.

Several mechanisms have been proposed underlying pathogenesis in X-ALD [33], mainly based on the impairment of mitochondrial redox homeostasis, GSH deficiency, and defective membranes’ function due to VLFCAs incorporation [4,33,34,35,36,37]. However, the pathophysiological mechanism of the disease remains currently poorly understood, limiting investigation of targeted therapeutic options.

The excess of VLCFAs, in particular the hexacosanoid acid C26:0, exerts various harmful activities in cells, the so-called lipotoxicity, triggering mitochondrial ROS production, GSH depletion, and decreased mitochondrial membrane potential [3,38]. A condition of oxidative stress, when associated with lipid dysregulation, may cause lipid peroxidation that, physiologically, is neutralized by a complex antioxidant repair system constituted by multiple enzymes working in concert with each other to maintain redox balance and protect cell membranes. This antioxidant defense system includes glutathione peroxidase 4 (GPX4), which is able to neutralize lipid peroxides by converting them to their corresponding alcohols; glutathione reductase (GR) that regenerates glutathione in its active form (GSH), essential for ROS scavenging; NAD(P)H:quinone oxidoreductase-1 (NQO1) that detoxifies quinones to hydroquinones maintaining the correct redox balance in the membranes [20]. If not detoxified, lipid peroxides can propagate the peroxidation to the adjacent fatty acids, inducing membrane permeabilization and fragmentation [39,40,41]. In addition, lipid peroxidation generates highly reactive aldehydes (e.g., 4-hydroxy-2-nonenale) that bind to specific amino acids further impairing protein function [42].

In X-ALD, tissues and plasma VLCFA accumulation is the main biochemical feature of the disease [43] and GSH depletion, together with increased lipid peroxidation products (TBARS, 15-HETE) have been found in the blood of patients [4,5,21].

In this study we analyzed the expression of GPX4, GR, NQO1, and GSH, the focal node of the endogenous lipid-targeted antioxidant response, in fibroblasts of patients with AMN, the late onset and slowly progressive form of X-ALD, and in cALD, the cerebral inflammatory demyelinating form of early childhood, with the main objective to understand if they were differently modulated in the two clinical forms of the disease. Our findings highlight different response profiles in cALD compared to AMN, with a consistent up-regulation of the enzymes responsible for the lipid protection (GPX4 and NQO1) in cALD, whereas the GSH-recycling enzyme GR was more responsive in AMN, where NQO1 was even significantly reduced.

The contribution of NQO1 and GPX4 in maintaining lipid homeostasis and preventing the lethal accumulation of oxidized lipids is essential and multifaceted under conditions of oxidative stress. Indeed, NQO1 belongs to the plasma membrane redox system (PMRS) and has a double role: it reduces CoQ_10_ to ubiquinol and regenerates the lipid antioxidant α-tocopherol by ascorbate [20]. GPX4, assisted by GSH and GR, besides detoxifying the lipid peroxides, is even a key regulator of lipids signaling [44,45]. This antioxidant lipid protection appeared to be dysregulated in fibroblasts of patients with AMN and cALD and, as a consequence, lipid peroxides accumulated in cells. Importantly, when we treated cells with NAC, we found a rebalance of the GSH levels and a significant decrease of the oxidized lipids overload, thus confirming the close relationship between tissue redox equilibrium and lipid homeostasis. Many redox-active drugs are able to re-balance redox cell homeostasis, and some of these have been found to halt the axonal degeneration in a mouse model of X-ALD [2]. The use of lipophilic and hydrophilic antioxidant combinations could expand the spectrum of therapeutic options in a disease where, currently, treatments are restricted to bone marrow transplantation [46] and hematopoietic stem cell gene therapy for cALD, whereas no cure is still available for the AMN phenotype [47]. 

Lipid peroxidation is a hallmark of ferroptosis, a new type of cell death occurring when “GSH-dependent” and/or “CoQ_10_-dependent” repair systems are compromised [10,11,39,48,49]. With this study we suggest a role for ferroptosis as pathogenic mechanism underlying X-ALD and propose lipid peroxidation as the missing link between VLCFA overload and oxidative stress.

## 5. Conclusions

Collectively, our findings show a different response to the oxidative damage in patients with AMN and cALD. In particular, we found that lipid peroxides accumulate in X-ALD fibroblasts, and ferroptosis-counteracting enzymes were dysregulated. Our study can provide new insights into the pathogenic events underlying X-ALD and be useful to identify novel disease targets leading to personalized antioxidant signatures. 

## Figures and Tables

**Table 1:** Patients’ characteristics.

Patient	Age at Skin Biopsy (Yrs)	Mutation	C26:0 (µM)	C26:0/C22:0 (µM)
AMN	56	[c.1661G > A; p.Arg554His]	2.62	0.062
cALD #1	9	[c.1817C > T; p.(S606L)]	3.47	0.144
cALD #2	3	[c.1817C > T; p.(S606L)]	3.20	0.094
Controls (range)	---	---	0.01–0.9	0.006–0.020

**Table 2:** qRT-PCR and Western blot results expressed as percentage respect to healthy subjects (Ctrls).

Gene Expression (qRT-PCR)					Protein Amounts (WB)			
Patient	GPX4 (%)	GCL (%)	GR (%)	NQO1 (%)	Patient	Gpx4 (%)	Gr (%)	Nqo1 (%)
AMN	98	74	290	30	AMN	69	201	67
cALD #1	195	105	89	266	cALD #1	121	122	95
cALD #2	221	113	105	362	cALD #2	151	142	131
Ctrls	100	100	100	100	Ctrls	100	100	100

**Table 3:** Enzyme activities and glutathione levels expressed as percentage respect to Ctrls.

Patient	Gpx4 (%)	Nqo1 (%)	SH (%)	GSH (%)
AMN	68	53	78	75
cALD #1	64	136	76	76
cALD #2	109	138	77	77
Ctrls	100	100	100	100

**Table 4:** Glutathione and proteins levels in NAC-treated patients expressed as percentage respect to untreated ones.

	SH (%)	GSH (%)	Gpx4 (%)	Gr (%)	Nqo1 (%)
Patient	+NAC	+NAC	+NAC	+NAC	+NAC
AMN	157	161	123	63	124
cALD #1	138	141	40	57	82
cALD #2	137	141	45	144	146
Untreated	100	100	100	100	100

## Data Availability

Not applicable.
